# Prognostic value of the seventh AJCC/UICC TNM classification of non-cardia gastric cancer

**DOI:** 10.1186/1477-7819-11-103

**Published:** 2013-05-20

**Authors:** Luigina Graziosi, Elisabetta Marino, Emanuel Cavazzoni, Annibale Donini

**Affiliations:** 1 Department of General and Emergency Surgery, Santa Maria della Misericordia Hospital, University of Perugia, Via Dottori, 06134, Perugia, Italy

**Keywords:** Gastric cancer, Seventh TNM classification, Sixth TNM classification, Overall survival

## Abstract

**Background:**

The TNM staging criteria for gastric carcinoma have seen numerous revisions, the most recent of which are reflected in the seventh edition AJCC TNM cancer staging manual.

**Methods:**

A retrospective evaluation of the sixth and seventh TNM classification of gastric cancer on a prospective database, regarding patients operated on for primary gastric cancer, was conducted. The end point of the study was prognosis evaluation in terms of overall survival.

Patients operated on for primary gastric cancer between September 2003 and March 2012 at our Department of Emergency and General Surgery, were consecutively retrieved in this study; a total of 114 patients were considered. Cardia gastric cancers, gastric lymphomas and gastrointestinal stromal tumors (GIST) were excluded. Median and mean follow-up periods were 22.5 and 27.7 months (range 15 days to 5 years). Both TNM6 and TNM7 were used to evaluate our patients. Overall survival and survival rates at different stages were analyzed using the Kaplan-Meier method and differences were determined using a log-rank test. Cox’s proportional hazard model was used to identify significant factors related to prognosis in a multivariate analysis.

**Results:**

Overall survival between the sixth and seventh TNM classification was not significantly different. Both the Kaplan-Meier analysis and the multivariate analysis showed that the major negative prognostic factor was lymphovascular invasion (*P* < 0.001 in the univariate analysis and *P* = 0.035 to 0.048 in the multivariate analysis). Stage distribution and stage-related survival changed from the sixth to the seventh edition, especially in T3 stage where median survival for the sixth edition was 720 days versus 1,200 days for the seventh edition. Moreover, differences were shown in the survival rate of N1 versus N2 stages within the seventh TNM.

**Conclusions:**

Even though further studies are needed in order to increase the number of patients studied, the seventh edition seems to provide a more accurate prognosis, especially regarding N1 and N2 tumors, showing that the most important prognostic factor is lymphovascular invasion.

## Background

Gastric tumor is a disease with one of the poorest prognoses, being the second cause of tumor-related mortality in the world. Five-year overall survival (OS) is 25% or less, especially in USA, Europe, and China [1,2]. Its incidence varies according to different countries: Asian countries have a high incidence of 50/100,000, whereas in the occidental countries incidence falls to 18/100,000 [3]. For these reasons, the staging system is continuously evolving.

The main tumor-staging system is the AJCC/UICC TNM classification in which three parameters are simultaneously evaluated: gastric wall or other organ involvement by the primitive tumor (T); lymph node involvement (N); presence of distant metastases (M). Between the end of 2009 and 2010, a new TNM edition was developed, becoming the seventh edition.

The major differences are: 1) lymph node involvement staging; 2) primitive tumor staging; 3) the exclusion from the gastric cancer staging of esophageal cancer with epicenter within 5 cm of the esophagogastric junction (EGJ) that also extends into the esophagus; 4) the exclusion of Mx and Nx definitions from this classification (Additional file 1).

One of the most important prognostic factors for patients with gastric cancer is lymph node involvement, and it is clear that standardized dissection patterns relate to patient survival rate [4]. This is the main reason why, in the seventh edition, the substantial changes pertain mainly to lymph node staging; however it does not define the minimum number of lymph nodes necessary [5] for optimum staging. The aim of the study was to conduct a retrospective evaluation of the sixth and seventh TNM classification of gastric cancer on a prospectively- collected database. The end point of the study was prognosis evaluation in terms of overall survival.

## Methods

A database was adopted in order to store all data needed. Data included demographic (age at diagnosis and sex), esophagogastroduodenoscopic (EGDS) description, CT scan description, surgical procedure (resection type and lymphadenectomy), Lauren grade, pathological features, post-operative outcome, pTNM6 and pTNM7 stages, chemotherapy and hyperthermic intraperitoneal chemotherapy (HIPEC) treatments and serum tumor markers at the diagnosis and follow-up.

All data were stored prospectively and survival was updated at one, three and six months from surgery and every six months thereafter for 10 years.

All patients were treated according to the principles of the Helsinki Declaration, and signed an informed consent form.

Patients operated on for primary gastric cancer between September 2003 and March 2012 at our Department of Emergency and General Surgery of Perugia, were consecutively retrieved in this study. Cardia gastric cancers, gastric lymphomas and gastrointestinal stromal tumors (GIST) were excluded; a total of 114 patients were considered. Follow-up for the entire study population was conducted until death or the cut-off date that we arbitrarily decided (March 2012).

Median and mean follow-up periods were 22.5 and 27.7 months (range 15 days to 5 years), respectively. The clinicopathologic features of the patients are listed in Table 1. Of the 114 patients who underwent surgery, 11 (9.6%) had received neo-adjuvant chemotherapy, 52 (46.6%) received adjuvant chemotherapy after surgery and 15 (13.2%) received HIPEC either as prophylactic (pT4a and/or positive cytology) or as therapeutic (only when peritoneal cancer index was < 6) treatment.

**Table 1:** Clinicopathologic features of the 114 gastric cancer patients

**Characteristics**	**N**	**%**
Sex		
female	45	40
male	68	60
Age 7	0.5 ± 11.19	(37 to 91)
Tumor location		
lower	54	47
middle	34	30
upper	5	4
stump	2	2
entire	5	4
middle/lower	12	11
upper/middle	2	2
Lauren grade		
intestinal	65	57
diffuse	45	39
mixed	4	4
Lymphovascular invasion		
positive	68	60
negative	46	40
Adjuvant chemotherapy	52	466
Neo-adjuvant chemotherapy	11	96
HIPEC	15	132
Gastrectomy	46	403
Gastric resection	66	579
Resection of the gastric stump	2	18
D1	29	254
D2	76	667
D3	9	79

The intravenous chemotherapy regimen administered included platinum chemotherapies in association with capecitabine and docetaxel or epirubicin, whereas for HIPEC, mitomycin in association with cisplatin were administered.

**Figure 1:**
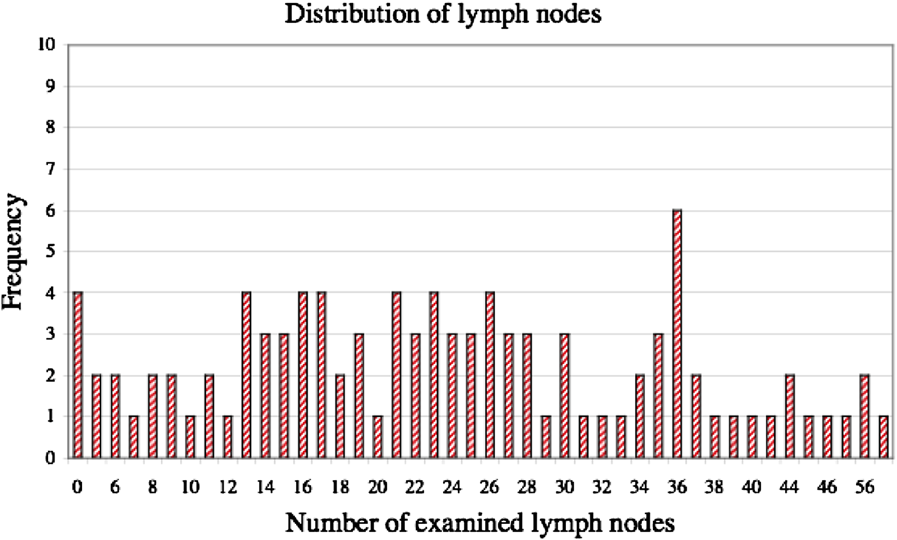
Frequency distribution of lymph nodes.

The pT category, pN category and stage of each tumor were classified according to both the sixth and seventh editions of the AJCC TNM staging system. Figure 1 shows the frequency distribution of examined lymph nodes for the entire cohort of patients.

### Statistical analysis

Overall survival and survival rates in different stages according to both the sixth and seventh TNM editions, were analyzed using the Kaplan-Meier method and the differences were determined using a log-rank test. In multivariate analysis, Cox’s proportional hazard model was used to identify significant factors related to prognosis.

A *P* < 0.05 was regarded as statistically significant. Graph Pad (©2013 GraphPad Software, Inc) Prism version 5.0 and SAS (© SAS Institute Inc.) statistical softwares were used to generate these analyses.

## Results

**Figure 2:**
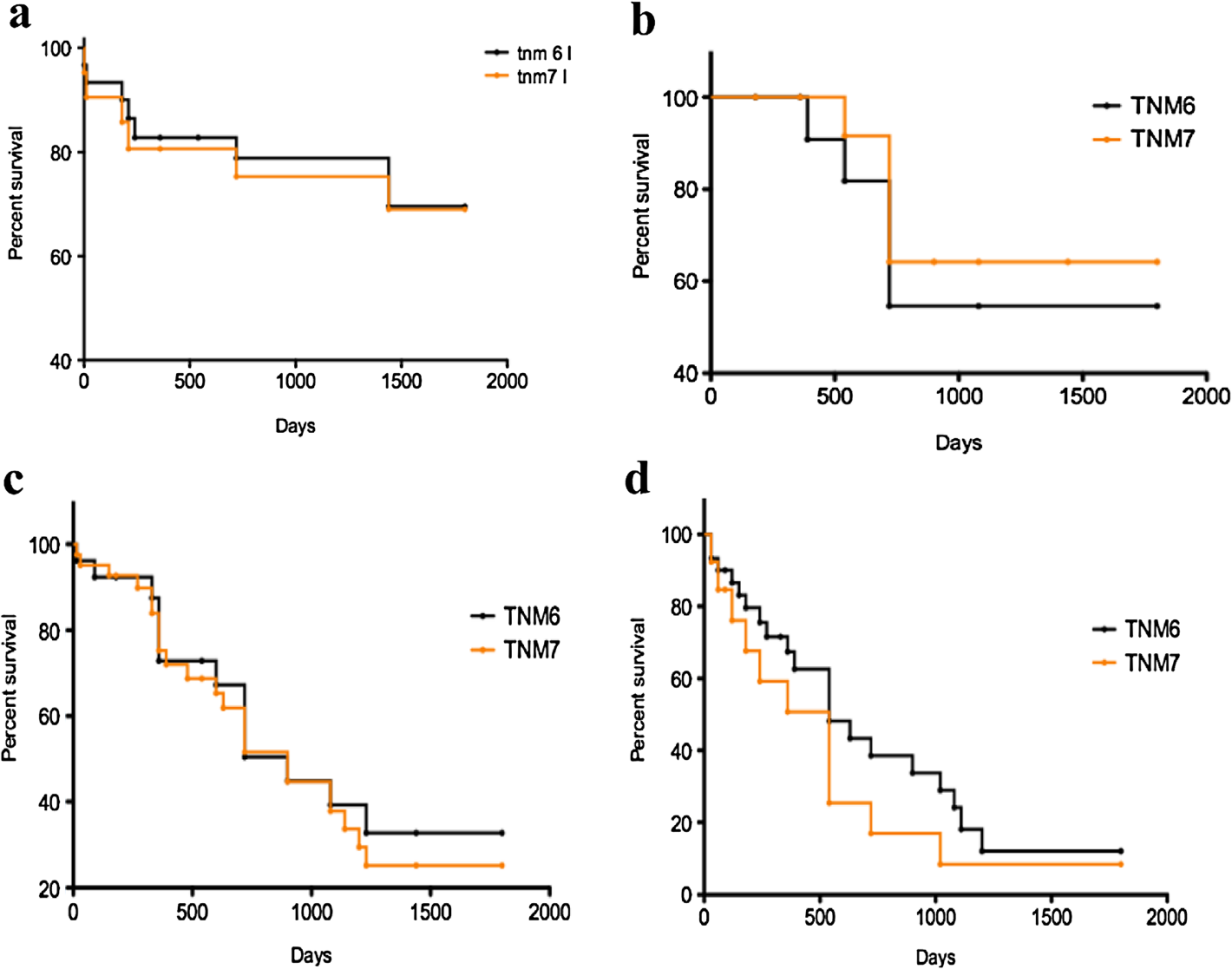
Survival comparison among different TNM editions in different stages: (a) stage I, (b) stage II, (c) stage III, (d) stage IV.

Comparisons of survival curves between patients in different TNM stages according to the sixth and seventh edition systems, are shown in Figure 2 (a-d). Additional file 2 and Additional file 3 also show how patients were divided into different pT, pN and stages according to both the TNM editions. Our tables show that patients in our database are mostly in an advanced disease stage using either the sixth or seventh TNM classification. The main differences between the two editions’ sub-classification apply to patients in stage IV. As a matter of fact, a higher number of patients fall in to stage IV according to sixth TNM due to the fact that in the revised edition, stage IV patients are the only ones with metastatic disease.

As shown, significant differences in prognosis could not always be observed between different TNM editions; early stages (I, II) have a higher survival, with a median of above five years for stage I and of two years for stage II.

**Figure 3:**
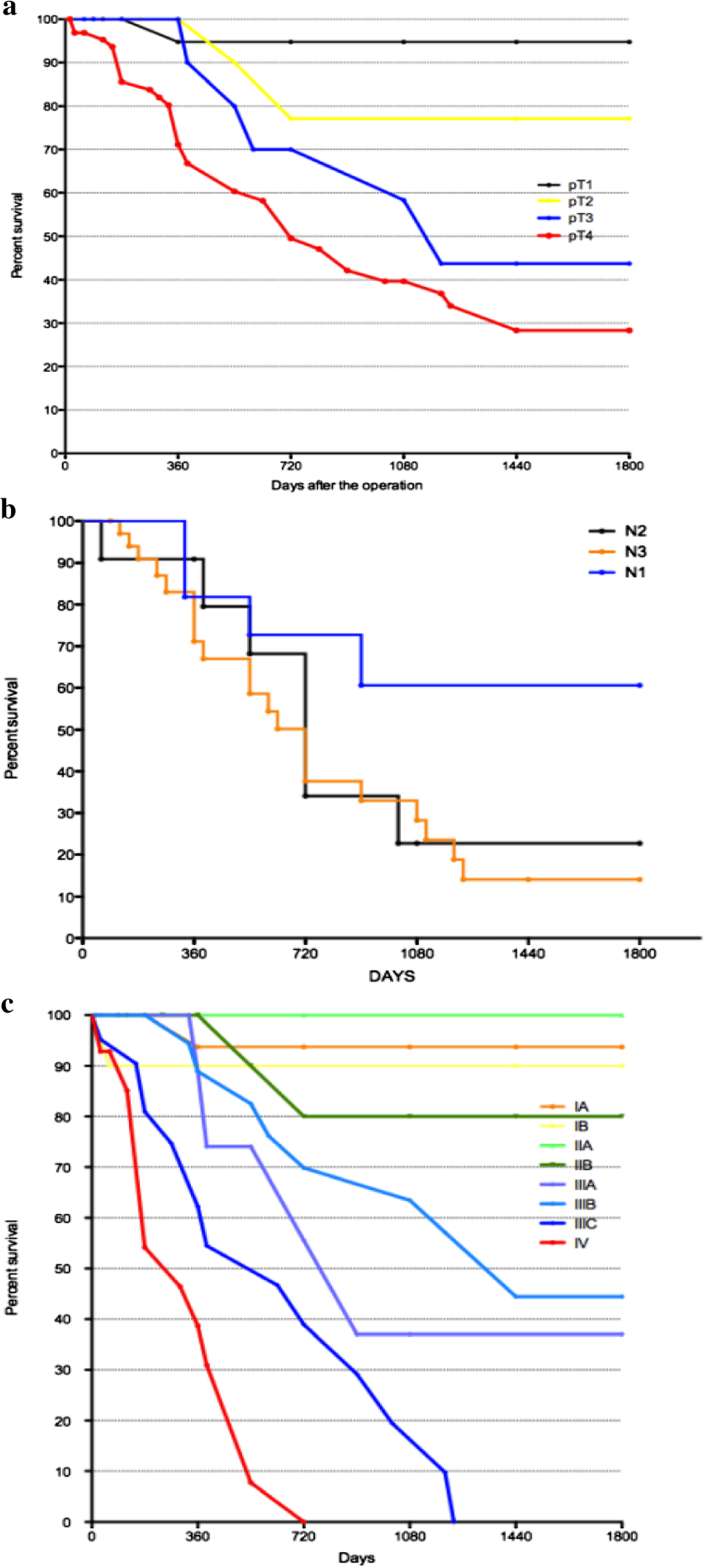
Survival curves of patients in different pT, pN and stages in the seventh TNM.

On the other hand, the prognosis in higher stages decreases significantly in both sixth and seventh TNM. Survival curves of patients in different pT categories, pN categories and stages, according to the seventh TNM edition system are shown in Figure 3 (a-c respectively). As shown, significant differences in prognosis could be observed in patients in pT2 and pT3 categories (*P* < 0.001) and in pN1 and pN2 categories (*P* < 0.005).

Five-year OS curve, according to the pT category, showed a significantly different survival rate among patients in T1 and T2 and T2 and T3, meaning that muscularis propria involvement and more specifically, subserosa involvement, are major negative prognostic factors.

Also the five-year survival curve according to the pN category, showed a significant change in prognosis between patients with one to two positive lymph nodes (N1) and patients with three to six positive lymph nodes (N2) (*P* < 0.005). Median survival was above 1,800 days and 720 days respectively.

**Figure 4:**
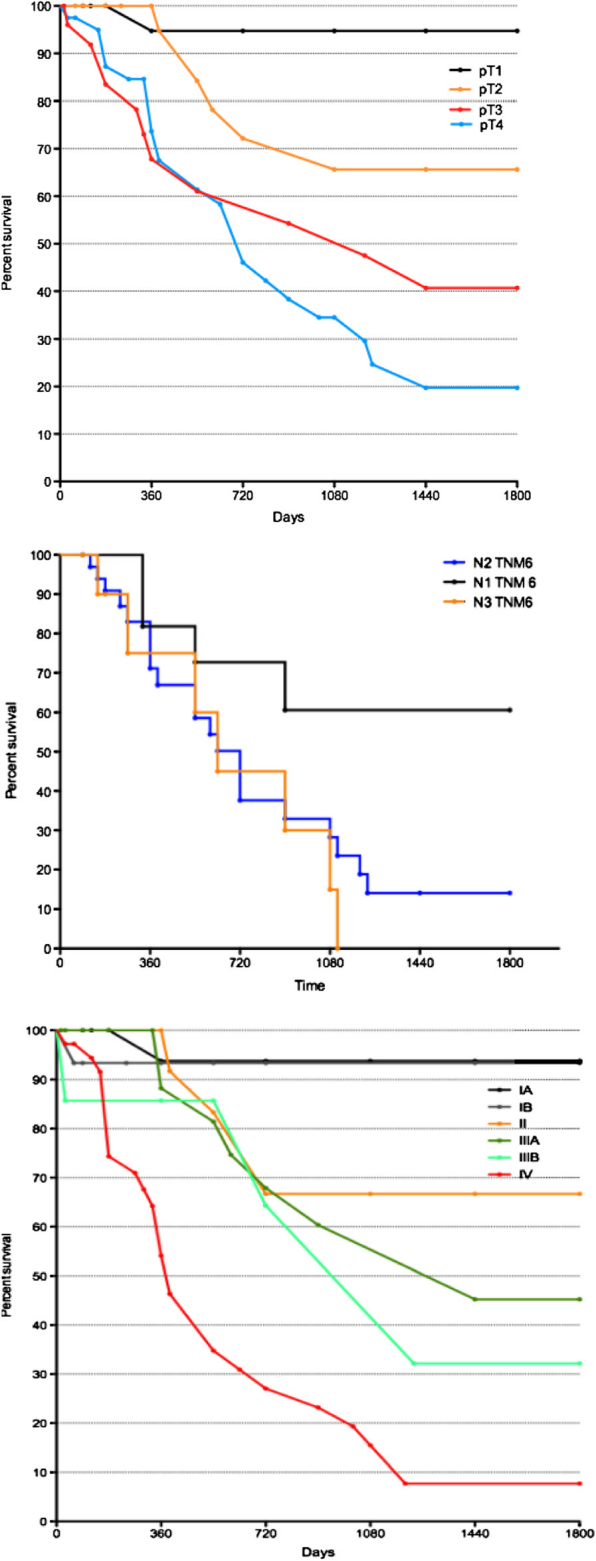
Survival curves of patients in different pT, pN and stages in the sixth TNM.

Comparing these data to those obtained with the sixth TNM edition, some differences occurred as shown in Figure 4. According to the sixth TNM edition, there is less homogeneity in the stages, and a lower five-year survival difference in the pT and pN categories.

To further investigate the validity of our study, we performed univariate and two-step multivariate analyses in which Cox’s proportional hazard model was used to identify significant factors related to prognosis (Additional file 4). Analyzing various different factors: sex, age, tumor location, Lauren grade, lymphovascular invasion, adjuvant therapy, neo-adjuvant therapy, HIPEC and TNM [6,7] staging, it is clear that the lymphovascular invasion status significantly correlates with survival (*P* < 0.001). Moreover, in the second step of multivariate analysis, lymphovascular invasion is confirmed to be an independent prognostic factor.

## Discussion

Accurate gastric cancer staging provides the means for appropriate treatment selection and defining prognosis. It is also the standard for reporting cancer incidence and outcomes. The TNM staging criteria for gastric adenocarcinoma have seen numerous revisions, the most recent of which are reflected in the seventh edition AJCC TNM cancer staging manual [6]. Previous sixth edition T categories T2a and T2b (denoting muscularis proprial and subserosal invasion, respectively) have been reclassified into T2 (muscularis proprial) and T3 (subserosal), respectively; this change reflects a significantly longer disease-specific five-year survival rate for tumors invading the muscularis propria compared with those invading the subserosa [7-9].

Upstaging from T2 to T3 places each tumor in a higher stage grouping for all stages. The previously categorized T3 category (serosal invasion) has now been changed to T4a, with the classification of adjacent structure involvement changed from T4 to T4b. No longer do T4b tumors denote stage IV disease by default; M1 disease is now characterized only by the presence of distant metastases and peritoneal washing-positive cytology. This is important as *en bloc* surgical resection for T4 tumors is considered a viable surgical strategy for potentially curative therapy.

In our study, we demonstrated a significantly different survival among patients in T1 and T2 according to pT categories, meaning that muscularis proprial invasion is a negative prognostic factor for survival. In addition, we showed significant survival discrimination between pT2 and pT3 groups, emphasizing the concept that subserosal involvement is a worse prognostic factor than serosal involvement.

Accordingly, we believe that the T stage categories in the seventh AJCC edition staging system have better prognostic value than the categories in the sixth edition.

In the sixth edition of AJCC TNM staging system, cases in which the number of metastatic lymph nodes were 1 to 6, 7 to 15 and ≥ 16 were classified as N1, N2 and N3, respectively. Of these, the N3 was classified as stage IV.

According to the seventh edition of AJCC TNM staging system which was newly revised, cases in which the number of metastatic lymph nodes are 1 to 2, 3 to 6 and greater than 7 are determined to be N1, N2 and N3, respectively. Of these, the N3 group was sub-classified; cases in which the number of metastatic lymph nodes are 7 to 15 and greater than 16, are determined to be N3a and N3b, respectively. However, because N3a and N3b are classified as the same TNM stage, the new classification system is not relevant for this group.

As described here, some cases were defined as the same TNM stage, although the range of number of lymph nodes involved was too wide. Despite the same TNM stage, the difference in the survival rate based on a specific cut-off point might reach a statistical significance as Kim has demonstrated in his work [10].

Although the sixth edition UICC TNM staging system is simple, reliable, and reproducible, for cases in which < 15 lymph nodes are examined, N stage may be incorrect because of stage migration. A method for bypassing this problem is to consider the ratio between metastatic and examined lymph nodes. On the other hand, in the seventh edition system, patients may be classified as N3 as long as the number of retrieved lymph nodes is more than seven, and thus, this revised edition system may reduce stage migration.

We demonstrated a large survival difference between N0 and N1 groups; as N1 denotes one to two involved nodes, this may suggest that prognosis is different for patients with a single metastatic node compared with two nodes. This lymph nodal subdivision is important in particular for patients with an early disease stage that could benefit from perioperative chemotherapy treatment to reduce postsurgical recurrences.

It also suggests that there may be subsets of N0 patients with poor prognostic features not captured by the current staging criteria. The different prognosis between the seventh AJCC N1 and N2 observed in our patients may have been influenced by the greater frequency of locally advanced gastric cancer patients due to the lack of screening tests, and an increasing incidence of proximal gastric cancer compared to that in Eastern populations.

Although some reports have shown no difference in survival rate between patients experiencing metastasis in one to three lymph nodes compared to those with four or more lymph nodes in early gastric cancer [11,12], comparison of five-year survival rate of gastric cancer by old and new UICC stage classification suggested that metastasis in more than four lymph nodes is a significant risk factor for recurrence.

Nio *et al.*[13] reported no difference in survival rate between stages N2 and N3 using the fifth UICC edition but did observe a significant difference in survival rate when N1 was divided into N1a (metastasis in one to three lymph nodes) and N1b (metastasis in four to six lymph nodes), supporting the subdivision of N1.

The UICC/AJCC TNM classification for gastric cancer is a manual containing the periodical promotion and modification about cancer staging. The seventh UICC/AJCC pN stage of gastric cancer is the latest edition for evaluating positive node metastases from gastric cancer, and has been validated to be more accurate than the previous edition of the pN stage for predication of the OS of patients after surgery. Actually, the comparatively elaborate pN stages of the seventh UICC/AJCC TNM classification for gastric cancer can significantly improve the prognostic precision of patients following curative resection. In our previous study, we demonstrated the seventh UICC/AJCC pN stage of gastric cancer was superior to the fifth/sixth UICC/AJCC pN stage.

We demonstrated in our multivariate analysis that the most influential factor for the prognosis of our patients, affected by locally advanced tumor, was lymphovascular involvement. For this reason, an accurate and extensive surgical lymphadenectomy with target chemotherapy could improve prognosis.

Although our study showed that the recategorized N staging system is more accurate than the traditional N staging system, further prospective studies would provide additional evidence supporting the use of a recategorized N staging system and metastatic lymph node ratio as a standard for the N staging of gastric cancer, especially when the number of retrieved lymph nodes is insufficient.

The biggest revision in the seventh AJCC edition is the division of sixth AJCC edition stage IV gastric cancer into stages IIB, IIIA, IIIB, IIIC, and IV. Sixth edition stage IV cases without distant metastasis (T1-4N3M0 or T4N1-2) are no longer classified as stage IV in the seventh AJCC edition system. Gastric cancer without distant metastasis has a good prognosis, so the necessity of a more detailed categorization has been disputed [14-18]. Using the sixth AJCC edition system, survival rates were significantly lower for T4N1-2M0 than they were for T1-3N3M0 groups and also for any T or any N M1 group compared to that of the T4N3M0 group.

In the present study, no significant difference was observed between seventh edition stages IA and IB or between stages IIA and IIB that represent early disease stages. In these groups there were a small number of patients. However, significant differences were observed between other subgroups (between IIB and IIIA, IIIB and IIIC).

The five-year survival rates were 80% for the seventh AJCC edition stage IIB and 40% for IIIA, 45% for the seventh AJCC edition stage IIIB and 0% for IIIC, which were more accurate prognoses than when these cases were categorized as stage IV in the sixth AJCC classification system and IIIA, IIIB, IIIC patients. We showed that the seventh AJCC edition might be more useful than the sixth edition for prognosis prediction and establishing adjuvant therapy for stage IV gastric cancer patients.

## Conclusions

Even though further studies are needed in order to increase the number of patients studied, the seventh edition seems to provide a more accurate prognosis, especially regarding N1 and N2 tumors, showing that the most important prognostic factor is lymphovascular invasion.

## Abbreviations

AJCC: American Joint committee on cancer; GIST: Gastrointestinal stromal tumors; HIPEC: Hyperthermic intraperitoneal intraoperative chemotherapy; OS: Overall survival.

## Competing interests

The authors declare that they have no competing interests.

## Authors’ contributions

GL carried out the data stored and analysis and drafted the manuscript. ME carried out the data stored and statistical analysis and drafted the manuscript. CE revised the manuscript and the English language. DA revised the manuscript and the English language. All Authors read and approved the final manuscript.

## Supplementary Material

Additional file 1 TNM classifications of the sixth and seventh editions are shown. Click here for file

Additional file 2 Seventh TNM edition patients’ subdivision. Click here for file

Additional file 3 Sixth TNM edition patients’ subdivision. Click here for file

Additional file 4 Univariate and multivariate analyses. Click here for file
