# Supplementary material for: Prognostic value of the seventh AJCC/UICC TNM classification of non-cardia gastric cancer
Source: World J Surg Oncol. 2013 May 20;11:103. doi: 10.1186/1477-7819-11-103 (PMC3686645; doi:10.1186/1477-7819-11-103)
Supplement: Additional file 1 — TNM classifications of the sixth and seventh editions are shown. [file 1477-7819-11-103-S1.pdf]

Table 1. TNM classifications of the sixth and seventh editions are shown.

			M0				M1
			N0	N1	N2	N3	
		Depth / No. of involved LN	0	1 ~ 6	7 ~ 15	16 ~	
M0	T1	Mucosa, submucosa	IA	IB	II	IV	IV
	T2a	Muscularis propria	IB	II	IIIA	IV	IV
	T2b	Subserosa					
	T3	Serosa	II	IIIA	IIIB	IV	IV
	T4	Adjacent structure	IIIA	IV	IV	IV	IV
M1			IV	IV	IV	IV	IV

			M0					M1
			N0	N1	N2	N3a	N3b	
		Depth / No. of involved LN	0	1 ~ 2	3 ~ 6	7 ~ 15	16 ~	
M0	T1	Mucosa, submucosa	IA	IB	IIA	IIB	IIB	IV
	T2	Muscularis propria	IB	IIA	IIB	IIIA	IIIA	IV
	T3	Subserosa	IIA	IIB	IIIA	IIIB	IIIB	IV
	T4a	Serosa	IIB	IIIA	IIIB	IIIC	IIIC	IV
	T4b	Adjacent structure	IIIB	IIIB	IIIC	IIIC	IIIC	IV
M1			IV	IV	IV	IV	IV	IV
