# Supplementary material for: Prognostic value of the seventh AJCC/UICC TNM classification of non-cardia gastric cancer
Source: World J Surg Oncol. 2013 May 20;11:103. doi: 10.1186/1477-7819-11-103 (PMC3686645; doi:10.1186/1477-7819-11-103)
Supplement: Additional file 2 — Seventh TNM edition patients’ subdivision. [file 1477-7819-11-103-S2.pdf]

Table 3. Seventh TNM edition patients' subdivision

	pN0(0)	pN1 (1-2)	pN2 (3-6)	pN3a (7-15)	pN3b(>15)	M1
pT1 (MSM)	IA 15.79% n= 18	IB 2.63% n=3	IIA: 0% n=0	IIB: 0% n=0	IIB: 0% n=0	
pT2 (MP)	IB 9.65% n=11	IIA: 0% n=0	IIB 2.63% n=3	IIIA: 1.75% n=2	IIIA: 0.88% n=1	
pT3 (SS)	IIA: 1.75% n=2	IIB: 0.88% n=1	IIIA: 3.51% n=4	IIIB 3.51% n=4	IIIB 1.75% n=2	
pT4a (Se)	IIB: 7.02% n=8	IIIA: 4.38% n=5	IIIB 3.51% n=4	IIIC: 4.38% n=5	IIIC: 6.14% n=7	
pT4b (Si)	IIIB 6.14% n=7	IIIB 4.38% n=5	IIIC 0.88% n=1	IIIC: 2.63% n=3	IIIC: 3.51% n=4	
M1						IV: 12.28% n=14
