# Supplementary material for: Prognostic value of the seventh AJCC/UICC TNM classification of non-cardia gastric cancer
Source: World J Surg Oncol. 2013 May 20;11:103. doi: 10.1186/1477-7819-11-103 (PMC3686645; doi:10.1186/1477-7819-11-103)
Supplement: Additional file 3 — Sixth TNM edition patients’ subdivision. [file 1477-7819-11-103-S3.pdf]

**Table 4. Sixth TNM edition patients' subdivision**

| | pN0 (0) | pN1(1-6) | pN2 (7-15) | pN3 (>15) | M1 |
|---|---|---|---|---|---|
| pT1 (M/SM) | IA: 16.67% n= 19 | IB: 2.63% n=3 | II 0% n=0 | IV: 0% N=0 | |
| pT2a (MP) | IB: 7.02% n=8 | II: 2.63% n=3 | IIIA: 2.63% n=3 | IV: 0% n=0 | |
| pT2b (SS) | IB: 3.51% n=4 | II: 3.51% n=4 | IIIA: 3.51% n=4 | IV: 1.75% N=2 | |
| pT3 (Se) | II: 6.14% n=7 | IIIA:8.77% n=10 | IIIB: 3.51% n=4 | IV: 6.14% n=7 | |
| pT4 (Si) | III A 7.02% n= 8 | IV: 4.38% n=5 | IV: 1.75% n=2 | IV: 4.38% n=5 | |
| | | | | | IV: 14.04% n=16 |
