# Supplementary material for: Prognostic value of the seventh AJCC/UICC TNM classification of non-cardia gastric cancer
Source: World J Surg Oncol. 2013 May 20;11:103. doi: 10.1186/1477-7819-11-103 (PMC3686645; doi:10.1186/1477-7819-11-103)
Supplement: Additional file 4 — Univariate and multivariate analyses. [file 1477-7819-11-103-S4.pdf]

Table 5. Univariate and multivariate analyses

		Global survival					
		Univariariate		Multivariate			
		HR (95% IC)	p value	HR (95% IC)	p value	HR (95% IC)	p value
SEX (female vs male)		0.63 (0.34-1.18)	0.148				
AGE		1.00 (0.98-1.03)	0.862				
TUMOR LOCATION (L)							
	M	0.60 (0.29-1.25)	0.175				
	U	1.20 (0.29-5.09)	0.806				
	LMU	1.78 (0.42-7.58)	0.438				
	ML	1.39 (0.53-3.65)	0.501				
	UM	-	-				
	STUMP	2.77 (0.64-11.95)	0.172				
LAUREN GRADE (Intestinal riferimento)							
	Diffuse	1.52 (0.85-2.73)	0.160				
	Mixed	1.29 (0.17-9.58)	0.804				
LYMPHOVASCULAR INVASION (yes vs no)		9.36 (3.67-23.88)	<0.001	3.18 (1.01-9.99)	0.048	3.08 (1.09-8.72)	0.035
NEOADIUVANTE (yes vs no)		0.94 (0.34-2.63)	0.909				
ADIUVANTE (yes vs no)		1.85 (1.02-3.36)	0.044	0.88 (0.47-1.64)	0.681	0.64 (0.33-1.24)	0.189
HIPEC (yes vs no)		1.32 (0.52-3.37)	0.566				
TNM_6 (IA riferimento)							
	IB	0.68 (0.06-7.51)	0.754				
	II	3.05 (0.56-16.71)	0.199				
	IIIA	5.56 (1.22-25.42)	0.027				
	IIIB	4.70 (0.66-33.48)	0.123				
	IV	18.31 (4.29-78.14)	<0.001				
TEST PER TREND TNM_6		1.88 (1.52-2.32)	<0.001	1.58 (1.22-2.05)	0.001		
TNM_7 (IA riferimento)							
	IB	0.70 (0.06-7.75)	0.773				
	IIA	-	-				
	IIB	1.67 (0.23-11.86)	0.610				
	IIIA	6.23 (1.12-34.56)	0.036				
	IIIB	5.15 (1.13-23.50)	0.035				
	IIIC	13.79 (3.06-62.19)	0.001				
	IV	33.11 (7.18-152.64)	<0.001				
TEST PER TREND TNM_7		1.82 (1.49-2.24)	<0.001			1.70 (1.33-2.17)	<0.001
